# Durability and Efficacy of Faricimab in Treatment-Resistant Retinal Edema Utilizing “Real-World” Dosing Regimens

**DOI:** 10.1155/2024/8583348

**Published:** 2024-11-04

**Authors:** Shravan V. Savant, James T. Kwan, Fina Barouch, Jeffrey Chang, David J. Ramsey, Jeffrey Marx, Gregory Blaha, Kendra Klein-Mascia

**Affiliations:** ^1^Department of Ophthalmology, Beth Israel Lahey Health, Burlington, Massachusetts, USA; ^2^Department of Ophthalmology, Tufts Medical Center, Boston, Massachusetts, USA

**Keywords:** faricimab, intravitreal injection, treat and extend, treatment-resistant edema

## Abstract

**Purpose:** To retrospectively analyze clinical outcomes of patients with “treatment-resistant” neovascular age-related macular degeneration or diabetic macular edema who were switched to intravitreal faricimab injections (IFIs) using a “real-world” treat-and-extend (TAE) protocol.

**Methods:** Seventy-one eyes from 62 patients receiving antivascular endothelial growth factor injections were evaluated before and after switching to IFI. Demographic and clinical data were collected. Primary endpoints were treatment interval extension and presence of intraretinal or subretinal fluid on spectral-domain optical coherence tomography (OCT) after switching to IFI. Secondary endpoints included best-corrected visual acuity, average OCT central subfield thickness, and presence of a pigment epithelium detachment and pigment epithelium detachment height.

**Results:** The average treatment interval after switching to IFI significantly increased from 37.6 ± 10.8 days to 45.2 ± 16.6 days (*p* = 0.0016). At the last follow-up, 35% of eyes were able to achieve a fluid-free status post-IFI. A chi-square test of independence validated this finding by showing a significant difference in the OCT findings trending towards less or no fluid on follow-up (*X*^2^ [3, *N* = 71] = 13.0705; *p* = 0.0003). The average central subfield thickness decreased from 327.2 ± 89.1 *μ*m to 294.8 ± 86.5 *μ*m (*p* = 0.0294). Best-corrected visual acuity, intraocular pressure, pigment epithelium detachment presence, and height had no significant difference after switching to IFI.

**Conclusions:** In “treatment-resistant” patients receiving anti-VEGF therapy for neovascular age-related macular degeneration or diabetic macular edema, switching to IFI in a “real-world” TAE protocol led to statistically significant improvements in treatment interval and retinal fluid on spectral domain OCT.

## 1. Introduction

The introduction of intravitreal anti-vascular endothelial growth factor (VEGF) injections revolutionized clinical outcomes in neovascular age-related macular degeneration (nAMD) and diabetic macular edema (DME), both potentially blinding retinal diseases. Despite these significant gains, a subset of patients sub-optimally respond to anti-VEGF therapy, either being unable to achieve a “fluid-free” state or treatment extension beyond a 1-month interval [1, 2]. In these “treatment-resistant” patients, a high treatment burden can be detrimental to quality of life, patient compliance and clinical outcomes [3, 4]. Increased clinic visits secondary to treatment-resistant patients increase the overall resource utilization and burden on the healthcare system. With the introduction of novel intravitreal injection agents targeting anti-VEGF and non-VEGF pathways, the expectation by physicians and patients alike is enhanced durability without sacrificing efficacy or safety.

In January 2022, faricimab (Vabysmo; Roche/Genentech, Basel, Switzerland) was approved by the Food and Drug Administration (FDA) for the treatment of nAMD and DME. It functions as a bi-specific monoclonal antibody that inhibits two angiogenic pathways: VEGF and angiotensin-2 (Ang-2). Dual inhibition is believed to have a synergistic effect and is thought to increase the durability of treatment response [5].

In clinical trial protocols, monthly loading doses are often employed prior to allowing for interval extension [6–9]. Notably, in phase 3 clinical trial protocols that led to the approval of faricimab in nAMD (TENAYA and LUCERNE) and DME (YOSEMITE and RHINE), at least four initial monthly loading doses were given before allowing for treatment extension [10, 11]. However, loading-dose protocols in the “real world” may not be feasible or aligned with patient preferences. It is possible that strict adherence to monthly loading doses is critical in achieving prolonged durability of treatment response, but understanding the efficacy with a less stringent treatment regimen may be helpful in guiding clinical management.

This retrospective study aims to characterize the clinical response to switching to IFI in “treatment-resistant” patients with nAMD or DME, as defined by either the inability to attain “fluid-free” status on OCT or the inability to extend the injection interval due to persistent or recurrent fluid.

## 2. Methods

This is a retrospective case series of patients treated with intravitreal injections for nAMD and DME by six retinal physicians at four clinical sites affiliated with a single tertiary academic center. Institutional review board and ethics committee approval was obtained, and all components of research followed the tenets of the Declaration of Helsinki. “Treatment-resistant” status was defined as persistent or worsening intraretinal fluid (IRF) or subretinal fluid (SRF) on OCT, or the inability to extend treatment intervals despite receiving monthly intravitreal injections. The decision to switch to IFI and the determination of follow-up intervals were at the discretion of the treating retinal physician. Intravitreal injections were administered in a treat-and-extend fashion that did not necessarily adhere to prespecified monthly loading doses. Deidentified clinical and demographic data were obtained from patients who received IFI from April 2022 to August 2023. Patients who had ophthalmic surgery (i.e., cataract surgery, glaucoma filtration surgery, and pars plana vitrectomy) during the collection interval or had less than 2 doses of intravitreal faricimab were excluded from the study.

Patient demographics and clinical data from the immediate patient visits preswitch to IFI as well as the last documented follow-up visit on IFI. Collected data included injection history (type and frequency of intravitreal agent) and best-corrected visual acuity (BCVA). Pre-IFI injection interval was defined as the interval immediately before switching to IFI on the prior agent. Post-IFI injection interval was the last recorded injection interval on the last documented visit. OCT parameters including central subfield thickness (CST), presence of pigment epithelial detachment (PED) based on clinician interpretation of the OCT, PED height, and presence of IRF or SRF were collected and analyzed. Fluid status was recorded in a binary fashion (i.e., IRF present or not and SRF present or not), and initial OCT interpretation noted by the treating physician was confirmed by secondary OCT review by another retina specialist (KKM and SS).

The primary endpoints were treatment interval extension and presence of “fluid-free” status on OCT following switch to IFI. Secondary endpoints included change in BCVA, average CST on OCT, presence of PED, and change in average PED height. All patients who had achieved a treatment interval greater than 4 weeks prior to switching to IFI were not reduced to a 4-week interval to undergo loading doses upon switch to IFI.

All statistics were performed in SPSS version 29 (IBM, New York, USA) and Microsoft Excel (Microsoft Inc., Washington, USA) and are reported as mean ± standard deviation where appropriate. Mean values regarding logMAR vision, CST values, etc. were comparatively analyzed with paired *T*-tests while a chi-square test of independence was used to assess the distribution of patients into various fluid states based on OCT findings. To ensure that follow-up intervals were not skewed by noncompliance, a Pearson correlation coefficient was calculated to correlate between recommended and actual follow-up intervals.

## 3. Results

### 3.1. Pre-IFI Switch Data

**Figure 1:**
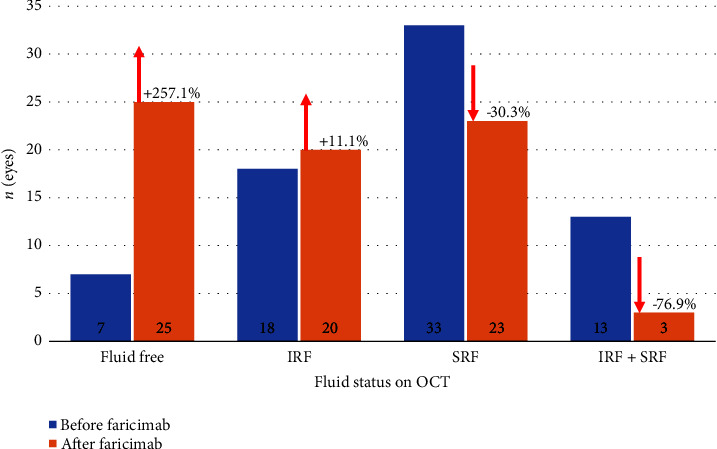
Optical coherence tomography fluid distribution before and after switch to IFI.

Seventy-one eyes from 62 patients obtained IFI during this 16-month observational period. The average patient age was 82 years old (range 53–97) and the majority were female (63.4%). Sixty-five eyes were treated for nAMD and six eyes were treated for DME. Patient ethnicity was nearly exclusively Caucasian (97.2%). On average, patients received 1.8 different intravitreal agents and had an average of 26.1 injections prior to switching to IFI. Seventy-one (100%) received either bevacizumab or aflibercept intravitreal injections prior to switching to IFI while 43 patients (60.6%) received both bevacizumab and aflibercept prior to switching to faricimab. Nine patients received bevacizumab only, 12 received aflibercept only, and seven received combinations of bevacizumab, aflibercept, brolucizumab, triamcinolone, and/or dexamethasone prior to switching to faricimab. Only patients in the DME cohort received intravitreal steroids. Full distribution of intravitreal therapy by indication is described in Table 1. The clinical reasons for switching to IFI included: refractory IRF or SRF (59.2%); inability to extend treatment interval (5.6%); or a combination of the two (35.2%). The average follow-up period was 218.7 ± 81.2 days, an average of 5.4 ± 2.2 follow-up visits. Prior to switching to IFI, the injection interval was an average of 37.6 days. Prior to switching to IFI, 25.3% of patients had IRF, 46.5% had SRF, and 18.3% had both IRF and SRF on OCT. The remaining 9.9% of patients had no fluid on OCT and were part of the subset of patients who switched to IFI to extend the treatment interval (Figure 1).

### 3.2. Post-IFI Switch Comparative Analysis

**Figure 2:**
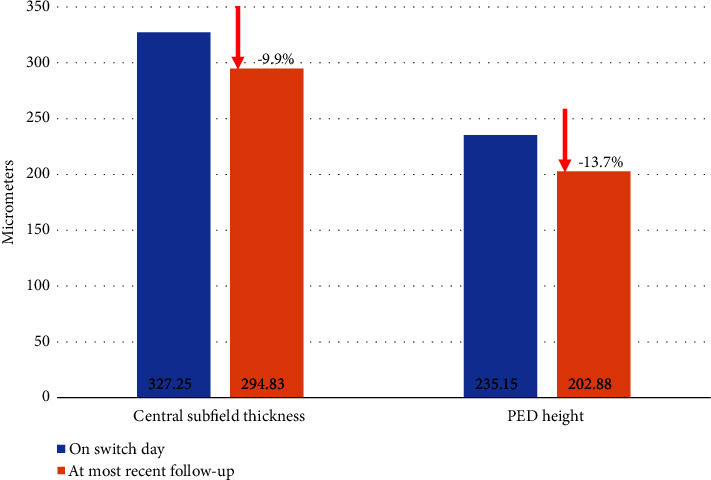
Central subfield thickness and PED height on optical coherence tomography at the time of switch to IFI and at the most recent follow-up.

**Figure 3:**
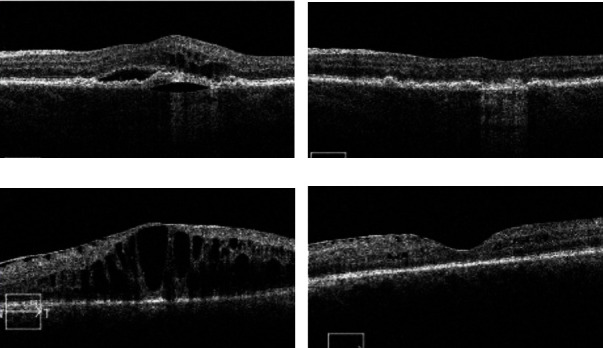
Review of OCTs pre-IFI and post-IFI. (a) A 93-year-old patient with nAMD who had tried aflibercept and bevacizumab with marked IRF, SRF, and a PED. (b) 4 months post-IFI in the same patient with collapsed PED and no IRF/SRF. Vision improved from 20/60 to 20/30. (c) 56-year-old patient with proliferative diabetic retinopathy and DME with epiretinal membrane who had trialed aflibercept and bevacizumab on a strict 4-week interval. (d) 6 months post-IFI in the same patient, with significant improvement in foveal contour and near resolution of IRF. Vision improved from 20/125 to 20/60.

There was a statistically significant increase in treatment interval from 37.6 ± 10.8 days to 45.2 ± 16.6 days (*p* = 0.0016) and a statistically significant reduction in average CST postswitch to IFI by 32 microns (327.2 ± 89.1 *μ*m versus 294.8 ± 86.5 *μ*m, *p* = 0.0294). There were no significant differences in the presence of PED pre- and postswitch to IFI (69.0% versus 75.0%, *p* = 0.4272), and although there was a reduction in PED height by 32 microns, this difference was not significant (*p* = 0.2440) (Figure 2). There was no statistically significant change in logMAR visual acuity (0.39 ± 0.33 [20/49] versus 0.44 ± 0.39 [20/55]; *p* = 0.4787) or IOP (13.0 ± 3.1 versus 12.6 ± 3.5; *p* = 0.5101) following switch to IFI. Twenty-five eyes were able to attain a fluid-free state compared to only 7 pre-IFI (a 257% increase; Figure 1). Eyes with both SRF and IRF decreased to only 3 patients compared to 13 patients pre-IFI. A chi-square test of independence showed that there was a significant difference in the OCT findings trending towards less/no fluid on follow-up (*χ*2 (3, *N* = 71) = 13.0705; *p* = 0.0003). To control for patient adherence to physician-recommended follow-up intervals, the Pearson correlation coefficient was calculated and found a significant correlation between recommended and actual follow-up intervals (*R* = 0.643, *p* < 0.001). For example, OCT changes seen in patients pre- and post-IFI can be seen in Figure 3.

Clinical outcomes were compared between patients with AMD versus DME on the day of switching and at the most recent follow-up to assess for differential response to IFI as seen in Table 2. For patients with DME, follow-up interval suggested immediately prior to switch day was significantly longer (50.5 ± 26.0 versus 36.4 ± 7.7, *p* = 0.002), IOP was higher on switch day (15.7 ± 4.6 versus 12.7 ± 2.8, *p* = 0.022) and at most recent follow-up (15.5 ± 3.2 versus 12.3 ± 3.5, *p* = 0.033), and though CST was thicker both on switch day and at most recent follow-up, change in CST was not significantly different (−15.5 ± 88.0 versus −34.0 ± 75.9, *p* = 0.575).

## 4. Discussion

DME and nAMD are potentially blinding retinal diseases and persistent IRF and SRF can lead to significant visual impairment and irreversible vision loss [12, 13]. Frequent and inflexible treatment intervals can negatively impact patient quality of life and compliance, especially when unplanned treatment extensions result in disease recurrence [14, 15]. Treat-and-extend regimens, which are popular with retinal specialists worldwide, tailor dosing intervals based on individual treatment responses in hopes of optimizing BCVA and CST outcomes while reducing treatment burden. Novel intravitreal injection agents, with robust drying capability and increased durability, may further reduce the treatment burden [16, 17]. Our analysis of previously treated patients with macular edema from age-related macular degeneration and diabetic retinopathy demonstrates that intravitreal faricimab significantly increases treatment interval and reduces CST. The heterogeneous nature of intravitreal agents tried prior to faricimab reflects the real-world complexities of treating recalcitrant retinal fluid, in which older agents (such as bevacizumab or aflibercept) are often tried first due to physician preference or insurance-mandated step therapy.

Faricimab, via dual inhibition of the VEGF and Ang-2 pathways, has shown improved durability of treatment response in patients with DME and nAMD. In TENAYA and LUCERNE, the pivotal phase 3 clinical trials leading to FDA approval, patients with treatment-naive nAMD received 4 initial monthly loading doses of faricimab prior to being extended into 8-, 12-, or 16-week fixed dosing arms in year 1. Treatment extension was based on disease activity criteria which included CST and BCVA criteria. In year 2, treatment intervals were determined by a modified treat-and-extend regimen whereby intervals were extended by 4 weeks or truncated by 4 or 8 weeks based on CST and BCVA criteria [10]. Similarly, the phase 3 YOSEMITE and RHINE trials, which investigated faricimab in treatment-naïve and previously treated DME, included a similar adjustable dosing regimen based on CST and BCVA measurements following prespecified monthly loading doses. These individualized dosing regimens were protocol-driven modified TAE regimens that can be seen in clinical practice [11, 18]. Clinical trials, however, do not always reflect clinical practice. Regarding patient population, novel agents are frequently used first in those with chronic, advanced, or treatment-resistant disease [19, 20]. Additionally, monthly loading doses are not always feasible due to a variety of factors, including the patient's desire to be extended, clinic scheduling constraints, or missed appointments. In the clinic, the decision to extend the treatment interval is likely less aggressive, with physicians less tolerant of extending with any retinal fluid or CST increases [21, 22]. When treatment extensions are granted, they may be in shorter intervals of one or 2 weeks rather than 4 weeks.

These factors, among others, may influence the results we see in daily practice from clinical trials. “Real-world” reports, although lacking the rigor of randomized control trials, are insightful in reflecting the clinical judgment and expected treatment responses in typical retina patients [23, 24]. One of the most comprehensive “real-world” studies, the TRUCKEE study by Khanani et al., retrospectively investigated the safety and efficacy of faricimab in real-world patients with previously treated and treatment-naive nAMD [25]. Clinical and anatomical outcomes including BCVA and CST were assessed prior to IFI, at follow-up after the first IFI, and after three administrations of IFI and did not require specific follow-up intervals or loading doses. Despite this, patients previously treated with aflibercept who were switched to faricimab maintained BCVA with a significant mean reduction in CST at a similar treatment interval prior to switching [25]. Other real-world studies regarding the use of faricimab have shown similar outcomes. Leung et al. reported that, at last follow-up, 24% of patients obtained fluid-free status on OCT, which is comparable to our rate of 35%. Their study had a more robust interval extension of approximately 2 weeks for patients in either the bevacizumab or aflibercept group compared to our extension of 1.08 weeks (7.6 days). This may be reflective of their dosing interval extension of 2 weeks per interval increase as opposed to our more conservative 1-week extension interval [26]. Watkins et al. developed a network meta-analysis from a systemic literature review of 26 large studies to indirectly compare the outcomes of a faricimab TAE protocol to other interventions. They concluded that faricimab had the best anatomic outcomes on OCT and subsequent lower treatment burden compared to aflibercept, ranibizumab, bevacizumab, and dexamethasone [27].

In our study, fewer than 10% of patients received three monthly loading doses, and the follow-up interval was determined by the treating physician. This reflects clinical practice where patients with a high treatment burden may be eager to extend follow-up or miss scheduled appointments. Despite the lack of loading doses, and with the average length of follow-up at the first, second, and third encounter after switching to IFI at 36.4, 39.5, and 41.0 days, respectively, significant reductions in CST were consistently observed (Figure 1). An average injection interval increase of every 38 days to every 45 days decreases the annual injection by approximately one to two shots per year compared to prior agents in these treatment-resistant patients. Although the 1-week interval extension in our treatment-resistant cohort was not consistent with the results seen in the pivotal faricimab clinical trials, it has the potential to be significant, both from a patient and system-based perspective.

Limitations in this study include the retrospective study design with a limited follow-up interval, small sample size, and lack of racial/ethnic diversity in the population studied. The study group had a higher amount of nAMD patients as opposed to DME. While the inclusion of DME patients improved the overall sample size and power of the study and allows us to draw upon the generalized utility of faricimab, the lack of homogeneity can allow for confounding variables. Future studies will likely benefit from separating treatment groups accordingly as well as from more OCT characteristic data extracted from these clinic visits, such as retinal pigment epithelium, outer retinal atrophy, and photoreceptor layer integrity. The clinical decision to switch a patient to a newer agent is likely to be made in the setting of recalcitrant or worsening fluid. Using the immediate preswitch visit as the comparison could introduce bias as patients may be more likely to be switched when they have increased fluid. Real-world studies, while possibly better reflecting clinical practice, are also subject to unintentional provider bias and unforeseen confounding. For example, the decision to switch being solely dependent on the provider's decision of necessity without a standardized criteria can skew the data based on provider preference. Minimal IRF and stable visual acuity may be enough for one physician to switch or reduce injection intervals while another would consider maintaining or extending based on functional stability. Our population largely represents a predominantly white population belonging to a unified, integrated health network, which may not be readily generalizable to other practice locations.

## 5. Conclusion

Intravitreal faricimab injections significantly increased treatment interval and reduced CST while maintaining vision in patients previously reported to have treatment-resistant nAMD and DME. These findings persisted despite no formal loading dose protocols when switching to faricimab and demonstrate its utility in real-world settings.

## Figures and Tables

**Table 1:** Injection characteristics at the time of switching by indication.

Prior injection history	AMD (*n* = 65)	DME (*n* = 6)	Total (*n* = 71)
Bevacizumab only	9 (13.8%)	0	9 (12.7%)
Aflibercept only	10 (15.4%)	0	10 (14.1%)
Bevacizumab OR aflibercept	65 (100%)	6 (100%)	71 (100%)
Bevacizumab AND aflibercept	44 (67.7%)	4 (66.7%)	48 (67.6%)
Other agents			
Brolucizumab	4 (6.2%)	0	4 (6.2%)
Steroids	0	2 (33.3%)	2 (33.3%)

*Note:* The “Other Agents” category includes combinations of bevacizumab or aflibercept with brolucizumab or steroids (triamcinolone or dexamethasone). Only patients in the DME cohort received intravitreal steroids.

**Table 2:** Assessment of differential response to IFI between AMD and DME.

	AMD (*n* = 65)	DME (*n* = 6)	*p*
Interval prior to switch	36.4 ± 7.7	50.5 ± 26.0	0.002
Interval at MRFU	45.8 ± 16.5	38.7 ± 17.0	0.317
IOP at switch	12.7 ± 2.8	15.7 ± 4.6	0.022
IOP at MRFU	12.3 ± 3.5	15.5 ± 3.2	0.033
VA at switch	0.38 ± 0.32	0.53 ± 0.35	0.271
VA at MRFU	0.43 ± 0.40	0.51 ± 0.26	0.624
CST at switch	315.6 ± 82.5	453.3 ± 57.5	< 0.001
CST at MRFU	281.6 ± 71.1	437.8 ± 114.9	< 0.001
ΔCST	−34.0 ± 75.9	−15.5 ± 88.0	0.575

Abbreviations: AMD = age-related macular degeneration, CST = central subfield thickness, DME = diabetic macular edema, IOP = intraocular pressure, MRFU = most recent follow-up, VA = visual acuity.

## Data Availability

The data that support the findings of this study are available from the corresponding author upon reasonable request.
